# Dynamic Reconstruction of Anal Sphincter with Camera Shutter Style Double-Opposing Gracilis Flaps

**DOI:** 10.1055/s-0043-1772755

**Published:** 2023-10-05

**Authors:** Allen Wei-Jiat Wong, Grace Hui-Min Tan, Frederick Hong-Xiang Koh, Min Hoe Chew

**Affiliations:** 1 Plastic, Reconstructive and Aesthetic Surgery Service, Department of General Surgery, Sengkang General Hospital, Singapore, Singapore; 2 Colorectal Service, Department of General Surgery, Sengkang General Hospital, Singapore, Singapore

**Keywords:** Fournier's gangrene, gracilis muscle, dynamic sphincter, anal sphincter reconstruction

## Abstract

Fournier's gangrene is a life-threatening infection which requires prompt recognition, early surgical debridement of unhealthy tissue, and initiation of broad-spectrum antibiotics. Relook debridement are usually performed until all the devitalized tissue has been removed. Involvement of the anal sphincter may result in significant morbidity such as permanent incontinence. Dynamic reconstruction of the anal sphincter has always been one of the holy grails in the field of pelvic reconstruction.

We demonstrate a new method of camera shutter style double-opposing gracilis muscle flaps that allows dynamic sphincteric function without the need for electrostimulation. The bilateral gracilis muscles are inset in a fashion that allows orthograde contraction of the muscle to narrow and collapse the neoanal opening. With biofeedback training, the patient is able to regain dynamic continence and return to function without a stoma. There was also no need for neurotization or microsurgery techniques to restore sphincteric function to the anus. The patient was able to reverse his stoma 14 months after the initial insult and reconstruction with biofeedback training without the use of electrostimulation.

## Introduction

Fourier's gangrene is an acute, rapidly progressive, and life-threatening infection in the genitalia and perineum region. Prompt recognition, early initiation of broad-spectrum antibiotics, and radical debridement of all infected tissue to obtain source control is important in the initial setting to give the best survival chance. 1 Nonetheless, the morbidity and mortality in these group of patients remain high.

Most of these patients will require some form of coverage subsequently, whether it is for skin coverage or obliteration of dead space, due to aggressive debridement that is usually performed to control the infection. Anal incontinence that results from anal sphincter involvement in Fournier's gangrene necessitates a colostomy. In such circumstances, reconstruction and restoration of the anal sphincter function is vital in order for the stoma to be reversed.

We present a case of Fournier's gangrene with anal sphincter involvement, who required a diverting colostomy. Bilateral gracilis muscle flaps were used for obliteration of dead space, and also to reconstruct the anal sphincter. Using a novel method of inset, the double gracilis flap is able to imitate the function of the anal sphincter. With biofeedback training alone, the patient is able to achieve continence, and was able to reverse his stoma.

## Idea

**Fig. 1:**
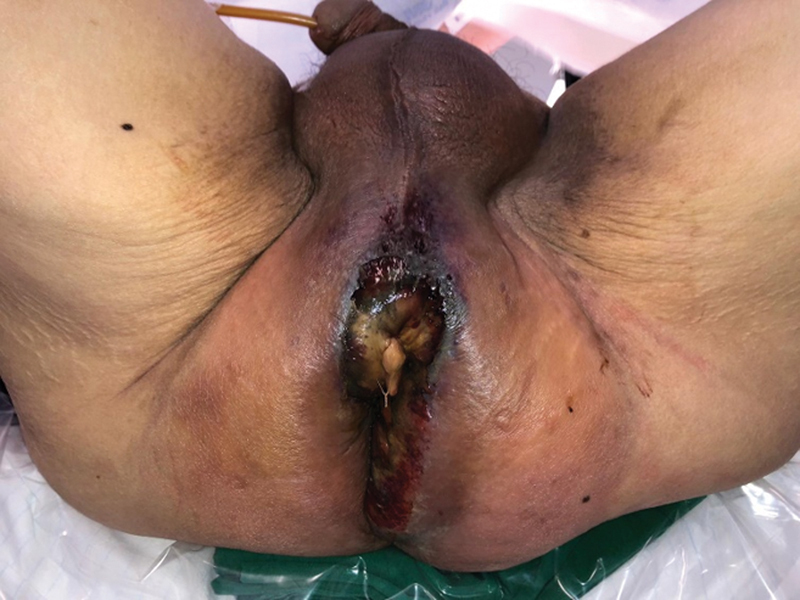
Initial presentation of the perineal region to the emergency department.

A 46-year-old male, with no significant past medical history, presented to our emergency department with fever and perineal tenderness on August 31, 2020. On examination, the patient appeared to be diaphoretic and lethargic, with a temperature of 38°C, tachycardia of 120 beats per minute, hypotensive at 79/52 mm Hg, and oxygen saturation was 100% on room air. There was a 5-cm indurated area over the perianal region at 11 o'clock position, associated with scrotal erythema and induration, with the epicenter of the infection diagnosed to be at the perianal region, involving the scrotum up to the penile base ( Fig. 1 ). He was attended to promptly by the general surgery team and the urology team in the emergency department. A diagnosis of septic shock secondary to Fournier's gangrene was made, and the patient was then brought straight to the operating theater after informed consent was taken.

**Fig. 2:**
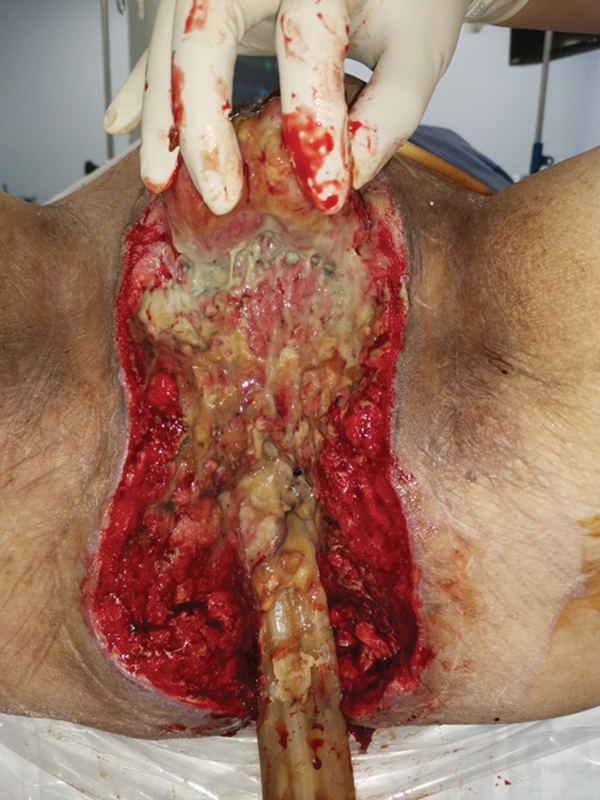
Hand holding bilateral scrotum up. Persistent soilage of wound.

**Fig. 3:**
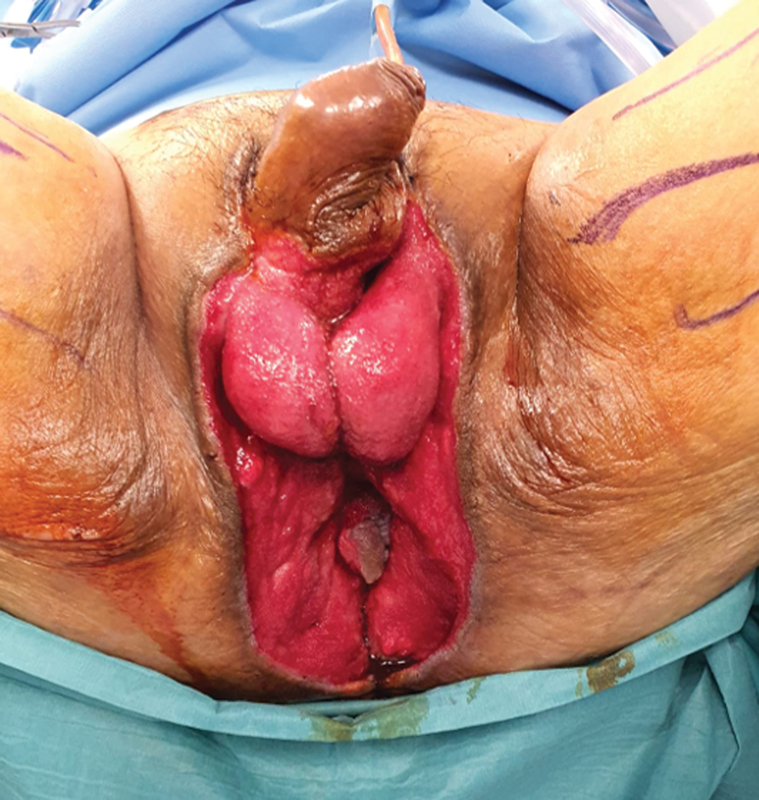
Resultant wound defect prior to flap reconstruction.

**Fig. 4:**
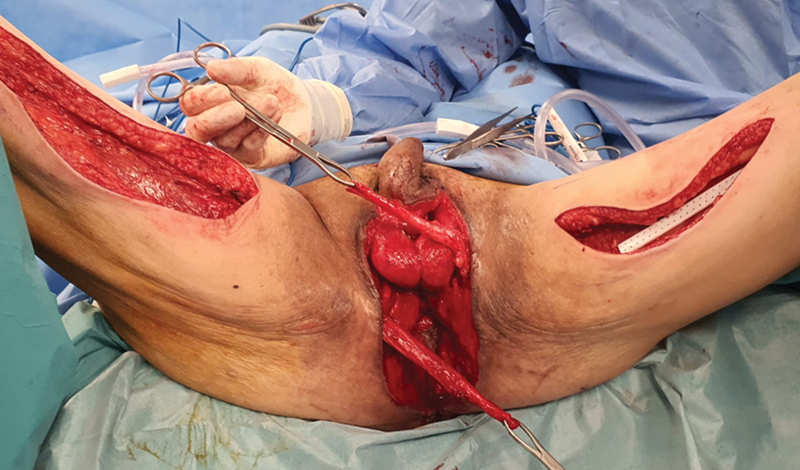
Harvest of bilateral gracilis flaps and inset of flaps.

**Fig. 5:**
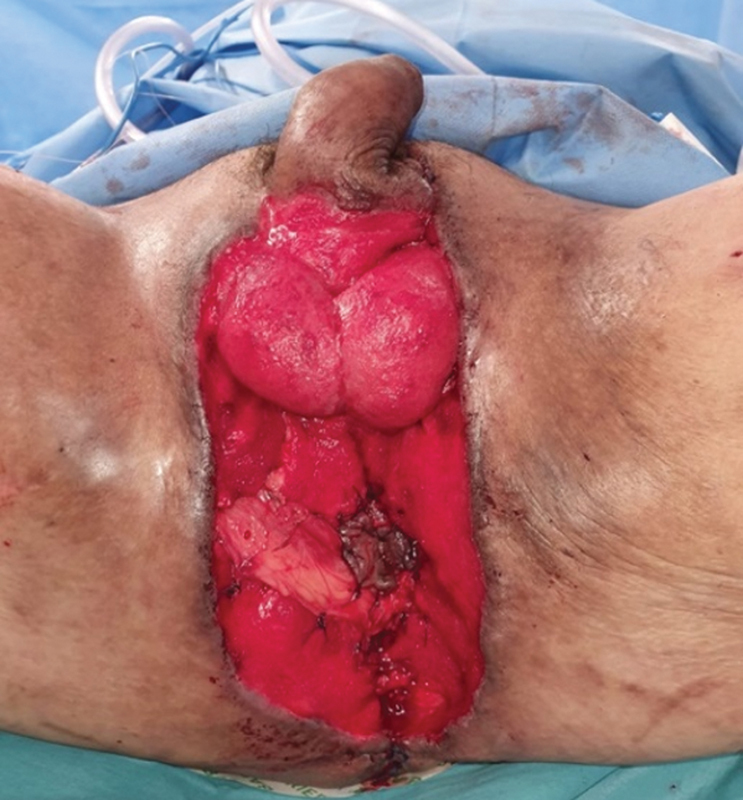
Final inset of flaps for the creation of neoanus.

**Fig. 6:**
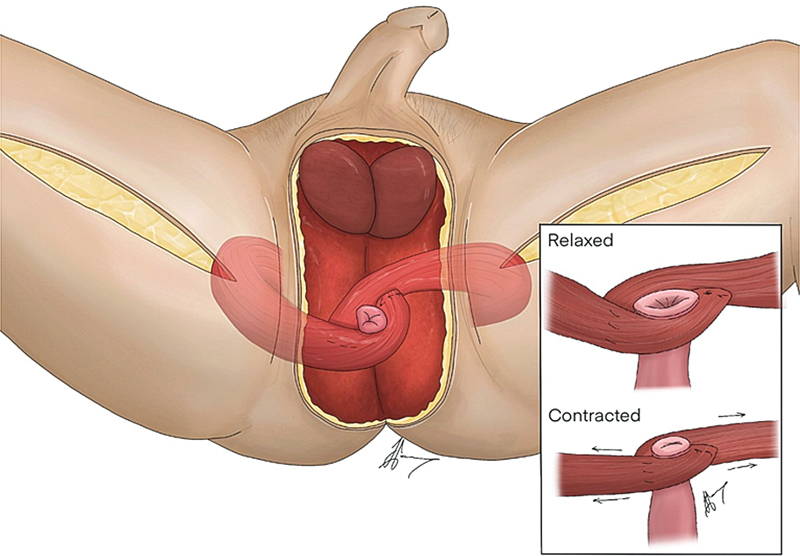
Illustration of technique.

**Fig. 7:**
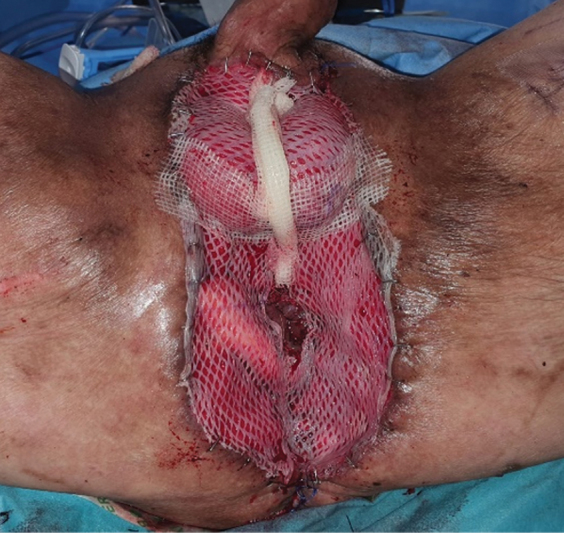
Skin grafts for resurfacing of raw surface.

All the unhealthy tissue was debrided and the patient was admitted to the intensive care unit as he required inotropic support. A relook debridement was performed the next day, and there was a 25 cm-by-25 cm area of dusky perineal skin with crepitus, and foul-smelling purulent discharge was noted from the 8 o'clock perineal region. All the necrotic tissue was excised and the pus was drained. Fecal soilage of the wound caused continued infection of the tissues surrounding the anus ( Fig. 2 ). To achieve source control, the colorectal surgeon had to debride all perisphincteric tissue, as this was the epicenter of infection. The patient was counseled for the likelihood of permanent incontinence, and thus together with the need to divert feces away from the perianal wound. The patient underwent a colostomy creation 2 weeks after the initial admission. During the workup, he was also diagnosed to have type II diabetes mellitus, and started on oral hypoglycemic agents. The tissue culture from the initial operation showed a mixture of *Streptococcus anginosus* and *Escherichia coli* . Subsequent debridement and wound cultures showed an evolving pattern of infection— *Klebsiella aerogenes* , *Pseudomonas aeruginosa* , and *Enterococcus faecium* . Intravenous augmentin was started for the patient on admission and changed to culture-directed antibiotics thereafter. After colostomy creation, fecal soilage of the wound was eliminated, but the patient still required another 2 weeks of repeat debridement before the wound was ready for definitive reconstruction. The resultant defect extended from the suprapubic region to the base of the penis, involving the bilateral scrotum, and to the perianal region involving the anus ( Fig. 3 ). The most critical defect was the exposed anus with lack of sphincteric muscles, Due to the necrotizing infection, all the sphincteric muscles had to be debrided by the colorectal surgeon (senior author), and resulting in incontinence,

About a month after the initial debridement (2 weeks after colostomy creation), definitive reconstruction was planned when the wound was clean with sufficient granulation tissue and tissue cultures were negative. The surgery was performed with the patient in a lithotomy position. After debridement and thorough washing of the wound, bilateral gracilis muscle flaps were harvested through medial inner thigh incisions, with preservation of the obturator nerves. The muscle flaps were then tunneled subcutaneously into the defect, so that they could abut the anus in a tension-free manner. The left gracilis was used to wrap the upper half of the sphincter, while the right gracilis was used to wrap the lower half of the sphincter. The distal tip of each gracilis flap was then anchored to the opposite gracilis flap, forming an aperture that is similar to that of a camera shutter ( Figs. 4 and 5 ). Thus, the double-opposing gracilis flaps formed a camera shutter, with the anal mucosa as its “aperture,” sprouted at the epicenter of the two muscle flaps ( Figs. 5 and 6 ). When both the gracilis muscles were activated, they would shorten and narrow the aperture of the anus, closing the anus and preventing leakage. The gracilis muscle flaps were anchored with vicryl 2/0 and vicryl 3/0. Split-thickness skin grafts were used to resurface the rest of the skin defect ( Fig. 7 ). Wound inspection on postoperative day 12 showed that the flap and the skin graft have contracted, forming a clear channel suitable for the passage of feces. During the patient's inpatient stay, his glycemic control was also maintained within an acceptable range.

After maturation of the double gracilis flaps and skin graft, a baseline anal sphincter manometry was performed for the patient. The mean resting pressure was 26.1 mm Hg, mean squeeze pressure was 29.1 mm Hg, and the maximum voluntary contraction was 32.4 mm Hg. On proctometrography, the rectal volume at initial sensation was 30 mL, first urge at 40 mL, and the maximal tolerable volume was 100 mL. The patient was then started on biofeedback training with the aim of using the double gracilis muscle flaps for dynamic restoration of the anal sphincter. To increase the closing tone of the neoanus, he was instructed to adduct his thighs to activate the double gracilis flaps. A combination of isometric and isotonic exercises of the thigh adductor muscle group were taught to the patient.

After 1 year of biofeedback training, he demonstrated dramatic improvement on the anal sphincter manometry. The final anal sphincter manometry performed for the patient showed the improved values of a mean resting pressure of 43.7 mm Hg, mean squeeze pressure of 101.6 mm Hg, and maximum voluntary contraction was 145.2 mm Hg. Rectosphincteric inhibitory reflex was noted to be present at 30 mL. The rectal volume at initial sensation was 48 mL, first urge at 99 mL, and the maximal tolerable volume at 160 mL. Although the resting and squeeze pressure and the rectoanal inhibitory reflex typically reflect sphincteric resting tone, it was unlikely to be due to the sphincters as they were debrided away by the colorectal surgeon. This improvement was likely to be due to the resting tone of the gracilis after 1 year of training with resultant neuroplasticity with hypertrophy. In view of the good results from the manometry and proctometrography, stoma reversal with anal continence was deemed feasible.

**Fig. 8:**
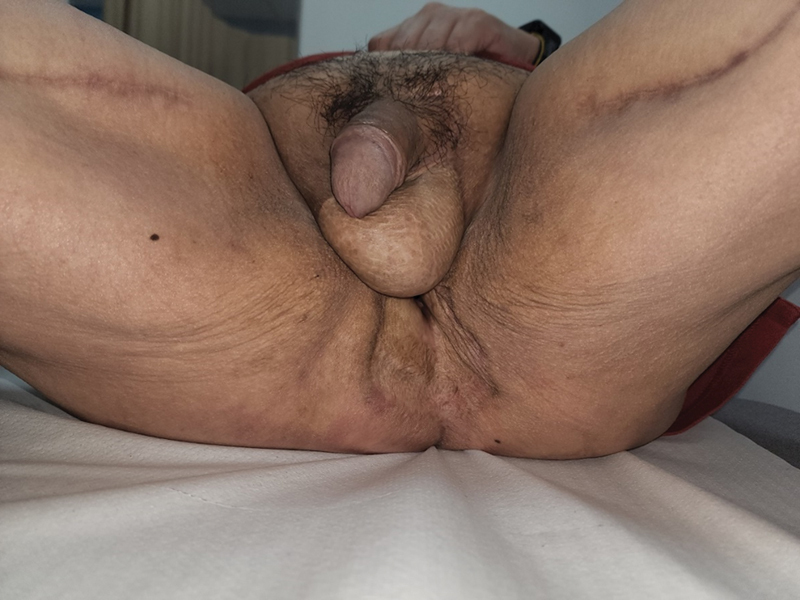
Twenty-four months postreconstruction of the anal sphincter—anal mucosa has contracted and wounds have all healed.

Fourteen months after the definitive reconstruction, the patient underwent reversal of stoma and dilatation of anal opening. He was able to pass flatus on postoperative day 1, and managed to pass motion on postoperative day 2. There was no evidence of fecal incontinence, and rectosphincteric inhibitory reflex was completely functional. The patient was followed up for a total of 12 months after stoma reversal, with maintenance of continence throughout, without any accidental discharge. With just one session of dilatation, the anal sphincteric function was well maintained without stenosis ( Fig. 8 ).

## Discussion

In cases of Fournier's gangrene with severe perineal involvement, the anal sphincters may be directly involved by the infection, necessitating the debridement of the structure, with the drastic morbidity of anal incontinence. Anal sphincter reconstruction is thus required in such cases but difficult to achieve. 2 The estimated percentage of patients requiring end-colostomy after radical debridement in Fournier's gangrene is approximately 15%. 3

Achieving a functional sphincteric reconstruction is still a holy grail in perineal reconstruction. Despite multiple attempts reported in the literature, a truly independent functional autologous sphincter without the use of external devices has still not been achieved. The use of unilateral or bilateral gracilis flap for anorectal reconstruction following abdominoperineal reconstruction, 4 trauma, iatrogenic causes, 5 or even anorectal malformation, 6 have been described in the literature. Gracilis is a muscle that exhausts quickly due to the fast-twitch fatigue-prone muscle fibers (type II muscle fibers). In the past, electrostimulation is commonly used after the graciloplasty to transform the gracilis muscle from type II to type I (slow-twitching fatigue-resistant fibers), which allows the gracilis muscle to work as a new sphincter and maintain a sustained contraction. 7 However, in most of these cases, they only achieved partial continence at best, with continued fecal leakage requiring diapers still. The success of conventional graciloplasty has been less than 50% mainly due to muscle fatigue and the inability of patients to voluntarily contract the transposed muscle. Gohil et al described the use of a single gracilis muscle wrapped around the anus in an “alpha,” “epsilon,” and “gamma” configuration, and showed that satisfactory continence was achieved in 76.4% of the patients in adynamic gracilis reconstruction. 7 The disadvantage of wrapping around the anus completely, was that it would exert a pulley effect, where the resultant force generated is centrifugal and could not collapse the anus to enforce continence. Rouanet et al used a gamma configuration for each gracilis muscle, and fixed both muscles to each other to create a double gracilis wrap. With electrostimulation as the next stage after the double gracilis wrap was performed, the study showed 5 out of 9 patients were continent for solids (55.6%). 8 However, this technique relied on electrostimulation to work.

In our novel technique, we describe a geometric way of inset that makes use of the orthograde contraction of the double gracilis flaps to narrow and collapse the neoanal opening. This is akin to the way a camera shutter closes, by “sliding and shuttering” the aperture close. The neurovascular pedicle of the gracilis muscle is carefully preserved so that the gracilis is still a functioning muscle and thus can be trained. Through biofeedback exercises, we instructed the patient to imagine adducting his thighs, and due to the geometry nature of inset, the contraction of the double gracilis muscles would be converted to a shuttering action on the anus, effectively closing the anus. A committed and compliant patient is necessary for the success of this technique, as our patient only achieved full continence after 1 year and 2 months of training. Since our technique does not rely on electrostimulation, the patient could reliably reverse his stoma without the need for long-term follow-up with electrical implants or devices. This technique can be thought of as a mechanical solution to an electrodynamic problem. There was also no need for microsurgery or neurotization procedures for this sphincteric reconstruction. The only “neurotization” involved would be the utilization of our cranial neuroplasticity during the biofeedback exercises.

With the inset of bilateral gracilis muscle flap in a double-opposing, camera shutter fashion around the anus, reconstruction of anal sphincter can be successful without the use of electrostimulation or complex microsurgery techniques. Intensive biofeedback training is required after surgery to achieve an acceptable anal resting and squeeze pressure before the reversal of stoma.
